# Imaging and Management of Scoliosis-Related Low Back Pain in Adolescents and Adults

**DOI:** 10.3390/diagnostics16203292

**Published:** 2026-10-10

**Authors:** Giulia Pacella, Raffaele Natella, Francesco Forte, Alfonso Forte, Luigi Dilorenzo, Giorgio Reverchon, Michela Bruno, Mario Brunese, Francesco Arrigoni, Corrado Caiazzo, Marcello Zappia

**Affiliations:** 1 Department of Medicine and Health Science “V. Tiberio,” University of Molise, 86100 Campobasso, Italy; 2 Fondazione Trotula de Ruggiero, 84121 Salerno, Italy; 3 Biomedical Research Centre, Gruppo Forte, 84124 Salerno, Italy; 4 Department of Life Sciences, Health and Health Professions, Link Campus University, 00165 Rome, Italy; 5 Department of Radiology, “St. Giuseppe Moscati” Hospital of National Relevance and High Specialty, 83100 Avellino, Italy; 6 Department of Precision Medicine, University of Campania “L.Vanvitelli”, 80138 Napoli, Italy; 7 Department of Biotechnological and Applied Clinical Sciences, University of L’Aquila, 67100 L’Aquila, Italy

**Keywords:** scoliosis, low back pain, pain generator, adolescent idiopathic scoliosis, adult spinal deformity, magnetic resonance imaging, Modic changes, basivertebral nerve ablation, EOS imaging, kinesiophobia

## Abstract

**Background/Objectives:** Low back pain (LBP) is a clinically relevant symptom in adolescents and adults with scoliosis, but its relationship with curve magnitude is inconsistent, and curve measurement alone therefore cannot explain symptom burden or direct pain management. This narrative review examines imaging findings and clinically plausible mechanisms of scoliosis-related LBP, considered separately for adolescent idiopathic scoliosis, adult idiopathic scoliosis, adult degenerative scoliosis and adult spinal deformity, and their implications for management. **Methods:** This narrative review was conducted and reported with reference to the SANRA quality domains. PubMed and the Cochrane Library were searched between June and July 2026, with database-specific search strings, search dates and record yields reported in full. Study selection was performed by the first author and reviewed by the co-authors, without independent duplicate screening or formal risk-of-bias assessment. **Results:** Curve magnitude is not a consistent independent predictor of pain burden, and pain distribution does not reliably follow curve laterality in adult degenerative scoliosis. We propose an author-derived and prospectively unvalidated seven-component framework comprising five candidate pain generators—facet-mediated, vertebrogenic/endplate, foraminal or lateral recess compression, sagittal malalignment with paraspinal overload, and sacroiliac overload—together with biopsychosocial pain modifiers and associated neural-axis abnormalities. Imaging may identify clinically plausible contributors but cannot establish pain causation in isolation; clinical concordance and, for selected interventions, supportive diagnostic procedures remain essential. Standing full-spine radiography, or low-dose biplanar acquisition where serial monitoring is required in adolescents, provides structural and alignment assessment, whereas MRI should be tailored to the clinical scenario and differs in purpose between adolescents and adults. Evidence supporting mechanism-directed interventions derives largely from non-scoliotic chronic LBP cohorts and is identified as such throughout. **Conclusions:** Imaging in scoliosis-related LBP should identify clinically concordant candidate pain sources rather than merely quantify deformity. Structural abnormalities are not synonymous with pain generators, and treatment selection should integrate imaging with clinical assessment and, when appropriate, supportive diagnostic procedures. The proposed framework and management pathway are author-derived and require prospective validation.

## 1. Introduction

Low back pain is a well-known leading cause of disability worldwide, with years lived with disability rising by 54% [1]. Scoliosis, defined as a lateral spinal deviation of at least 10 degrees on standing coronal radiography with obligate vertebral rotation, is among the most prevalent structural conditions associated with it, affecting 0.47–5.2% of the pediatric population [2].

This review distinguishes four populations, because of different etiology, natural history, and evidence base. Adolescent idiopathic scoliosis (AIS) denotes a coronal curve of ≥10° with vertebral rotation, of unknown cause, presenting between the age of 10 years and skeletal maturity. Adult idiopathic scoliosis (AdIS) denotes a curve of adolescent onset persisting into adulthood, on which degenerative change is subsequently superimposed. Adult degenerative scoliosis (ADS) denotes a curve arising de novo after skeletal maturity in a previously straight spine, through asymmetric disc and facet degeneration, and is typically lumbar. Adult spinal deformity (ASD) is a broader operative-planning construct encompassing coronal and sagittal malalignment of any cause and is the population in which the SRS–Schwab classification and age-adjusted alignment targets were developed. Where a cited study addresses one of these populations specifically, this is stated; where evidence is extrapolated across populations, or from general chronic LBP cohorts in which scoliosis was not an inclusion criterion, this is indicated explicitly and is not presented as scoliosis-specific efficacy evidence.

Available evidence does not support that deformity magnitude drives pain or that the Cobb angle is a sufficient or consistently independent predictor of pain burden. In fact, multivariable analyses in adolescents have generally failed to show an independent association between curve magnitude and pain intensity [3,4,5], while in ADS, pain drawings show no reliable association between the side of the convexity and the side of pain, which is often central and lumbar regardless of coronal deformity [6].

In a 50-year follow-up cohort, untreated late-onset idiopathic scoliosis was associated with more chronic back pain than in matched controls, but most affected patients described pain as little or moderate and remained functional at a mean age of 66 [7]. These findings should be interpreted as natural-history evidence rather than as proof that curve magnitude determines pain severity.

As a clinical consequence, if curve magnitude alone does not explain symptoms, measuring the curve alone cannot direct pain treatment. The diagnostic task therefore should identify a clinically plausible pain source, as imaging can identify phenotypes that are anatomically compatible with specific mechanisms and therapeutic targets. However, structural abnormalities are common and are not synonymous with symptom causation; therefore, imaging findings must be interpreted in conjunction with pain distribution, examination, and, for selected interventions, supportive diagnostic procedures, such as diagnostic blocks. These procedures are described here as supportive rather than confirmatory because single diagnostic blocks are subject to false-positive and false-negative responses and can strengthen, but not establish, the attribution of pain to a given structure [8].

Our group recently proposed an imaging-based framework for chronic LBP in the general population [9]. Carried over from that work are three elements: the general principle that an MRI phenotype should be matched to an anatomical target and, where a procedure is contemplated, to a supportive diagnostic step; the operational definitions of the radicular and non-radicular phenotypes; and the technical descriptions of the individual image-guided procedures. What is new to the present review is specific to the scoliotic spine: the evidentiary basis for the dissociation between curve magnitude and pain burden; the coronal and sagittal biomechanics through which deformity may generate asymmetric posterior-element, foraminal and paraspinal loading; the explicit separation of the four scoliosis populations defined above; age-dependent imaging strategy, including neural-axis abnormalities in AIS and cumulative radiation exposure during serial monitoring; and the distinction between deformity-directed and LBP-directed treatment, which has no counterpart in the non-scoliotic framework. The framework proposed here is therefore an extension rather than a restatement, and its scoliosis-specific components have not previously been published by this group. Two prior publications by overlapping author groups [9,10] are cited in the interventional sections; both are declared in the Conflicts of Interest statement.

Accordingly, this review examines the principal potential pain generators and other clinically relevant contributors to scoliosis-related LBP across the four populations defined above, their imaging correlates where applicable, and how imaging should be integrated with clinical and diagnostic-procedural findings to guide management. Curve classification, deformity progression, and surgical technique are considered only when directly relevant to LBP; comprehensive scoliosis correction is beyond the scope of this review [11,12].

## 2. Methods

This narrative review was conducted and reported with reference to the quality domains proposed by the Scale for the Assessment of Narrative Review Articles (SANRA), including justification of the review’s importance, explicit statement of aims, description of the literature search, appropriate referencing of key statements, scientific reasoning, and presentation of relevant data [13].

Searches were performed in PubMed (MEDLINE) and the Cochrane Library between 12 June and 24 July 2026 and were re-executed on 28 September 2026 to verify reproducibility; the strings and record yields reported below refer to the verification run. No language restriction was applied.

The core PubMed string was: (scoliosis [tiab] OR “spinal deformity” [tiab]) AND (“low back pain” [tiab] OR “back pain” [tiab] OR “lumbar pain” [tiab]), limited to 2018–2026, yielding 852 records. Three thematic strings were applied within the same concept structure to organise screening: an imaging string, adding (imaging [tiab] OR MRI [tiab] OR “magnetic resonance” [tiab] OR radiography [tiab] OR radiographic [tiab] OR EOS [tiab] OR Modic [tiab] OR facet [tiab] OR “foraminal stenosis” [tiab] OR “sagittal alignment” [tiab]), yielding 399 records; a management string, adding (bracing [tiab] OR brace [tiab] OR exercise [tiab] OR rehabilitation [tiab] OR physiotherapy [tiab] OR “radiofrequency ablation” [tiab] OR basivertebral [tiab] OR injection [tiab] OR fusion [tiab] OR surgery [tiab] OR surgical [tiab] OR conservative [tiab]), yielding 633 records; and a biopsychosocial string, adding (kinesiophobia [tiab] OR catastrophizing [tiab] OR “fear avoidance” [tiab] OR anxiety [tiab] OR depression [tiab] OR “quality of life” [tiab] OR psychosocial [tiab]), yielding 193 records. These three sets are overlapping subsets of the core set and were not summed.

The Cochrane Library was searched with the equivalent concept blocks: (scoliosis OR “spinal deformity”):ti,ab,kw AND (“low back pain” OR “back pain” OR “lumbar pain”):ti,ab,kw, limited to January 2018–December 2026, returning 183 records: one Cochrane Review, no Cochrane Protocols, and 182 CENTRAL trial records. The single Cochrane Review identified was retrieved in full and included [14]. CENTRAL records were screened to identify randomised trials not already retrieved through PubMed, and largely duplicated the PubMed yield. That a search of the Cochrane Library across nine years returned a single relevant systematic review is itself informative, and reflects the limited availability of high-certainty, scoliosis-specific evidence on low back pain that motivates the narrative format adopted here.

Duplicates between the two databases, and between the thematic subsets, were removed manually on the basis of title, first author, journal and DOI before screening. Records were screened on title and abstract, and retained records were assessed in full text. Reference lists of retained reviews and key articles were screened to identify landmark trials, natural-history cohorts, classification studies and society guidelines published before 2018, which were eligible irrespective of date. Sixty-three publications were included in the final synthesis.

Screening and selection were performed by the first author and subsequently reviewed by the co-authors; disagreements were resolved by discussion. Independent duplicate screening was not performed, and no formal risk-of-bias instrument or certainty-of-evidence framework was applied. Because this is a narrative rather than a systematic review, selection was guided by relevance and evidentiary value rather than by predefined eligibility thresholds, and the resulting evidence base cannot be assumed to be exhaustive or free of selection bias; this is stated as a limitation, and the design and applicability of the evidence supporting each principal conclusion are reported in the evidence table at the end of Section 6.

We included peer-reviewed original studies, systematic reviews, meta-analyses, randomized trials, larger observational studies, and relevant professional or society guidelines addressing the imaging, pathophysiology, clinical determinants, or management of scoliosis-related LBP in adolescents and adults. Preprints, conference abstracts, single case reports, and very small uncontrolled case series without specific methodological, mechanistic, or imaging relevance were excluded. Where scoliosis-specific evidence was limited, selected studies and guidelines from the broader LBP literature were considered to inform general imaging principles and mechanism-directed treatment selection; such evidence was not interpreted as scoliosis-specific efficacy evidence and is labelled as extrapolated in that table.

Because AIS studies frequently report the broader endpoint of “back pain” without anatomical localization [15], such evidence was not interpreted as direct evidence of LBP unless lumbar pain was explicitly defined. Studies using broader back-pain outcomes were included only when relevant to pain burden, determinants, or management. Studies confined to childhood, early-onset, or juvenile scoliosis were not used to support age-specific recommendations for adolescents or adults; a randomized controlled trial protocol [16] was included only to highlight an evidence gap and did not underpin any recommendation.

**Figure 1:**
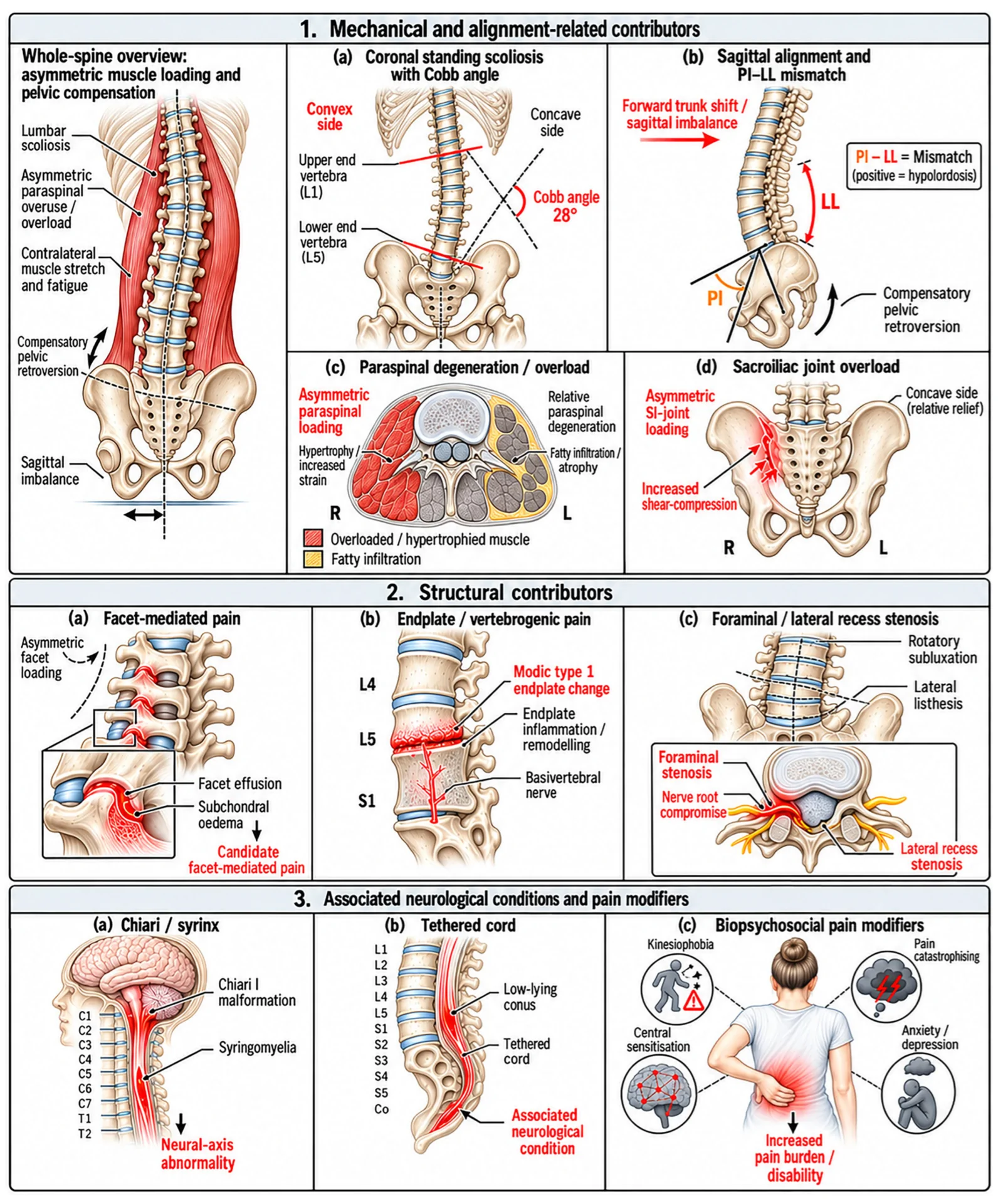
Conceptual overview of contributors to scoliosis-related low back pain. Author-proposed conceptual model, not a validated clinical algorithm. Mechanical and alignment-related factors include coronal and sagittal deformity, paraspinal degeneration and overload, and sacroiliac joint overload; structural contributors include facet, endplate/vertebrogenic, and foraminal or lateral recess abnormalities; associated neurological conditions and biopsychosocial pain modifiers are shown separately because they are not pain generators in the same sense. Red arrows, labels, and highlighted regions indicate the direction or site of the mechanical loading, compression, neural involvement, or other abnormality specified in each panel; black arrows and dashed lines indicate anatomical relationships, compensatory movements, or alignment/measurement axes. In the paraspinal muscle panel, red shading indicates overloaded/hypertrophied muscle and yellow shading indicates fatty infiltration. Imaging findings identify plausible contributors but do not establish pain causation; clinical concordance remains essential. The model requires prospective validation. Original figure created by the authors; final graphical rendering produced with the Canva app in ChatGPT (Canva Pty Ltd., Sydney, Australia; ChatGPT latest version in July 2026, OpenAI, San Francisco, CA, USA) on the basis of author-drafted layouts.

**Figure 2:**
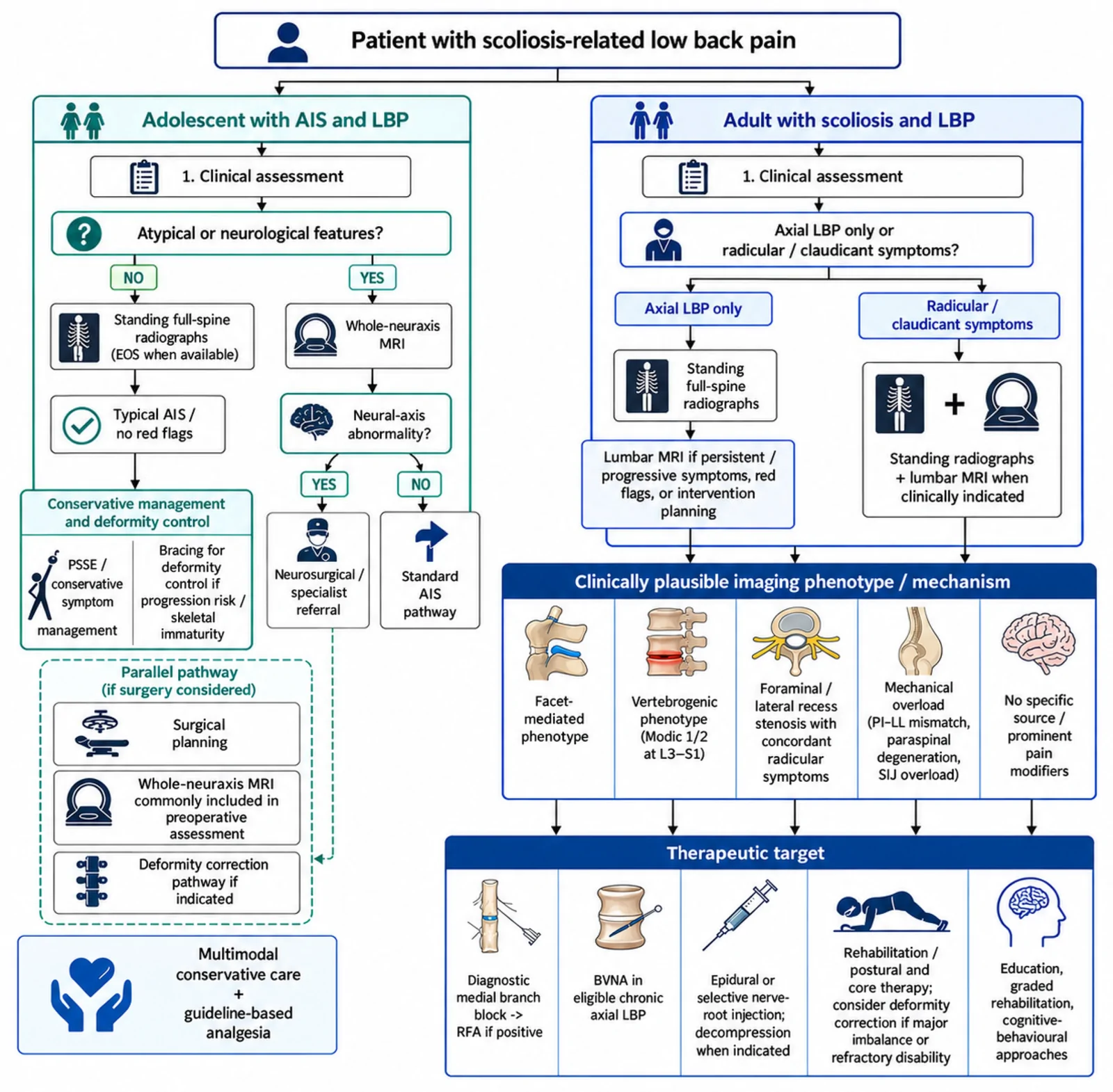
Author-proposed conceptual pathway from clinical presentation and imaging phenotype to therapeutic target in scoliosis-related low back pain. This is a conceptual model derived by the authors from the literature reviewed, not a validated or externally endorsed clinical algorithm, and it has not been prospectively tested. Imaging findings identify clinically plausible contributors but do not establish symptom causation; treatment selection requires clinical concordance and, for selected interventions, supportive diagnostic procedures. Population applicability differs between adolescents and adults and is indicated within the figure. Original figure created by the authors; final graphical rendering produced with the Canva app in ChatGPT (Canva Pty Ltd., Sydney, Australia; ChatGPT latest version in July 2026, OpenAI, San Francisco, CA, USA) on the basis of author-drafted layouts.

The conceptual content of Figure 1 and Figure 2 was drafted by the authors; the final graphical rendering was produced with Canva (https://www.canva.cn/en/?display-com-option=true (accessed on 1 July 2026); Canva Pty Ltd., Sydney, Australia), assisted by ChatGPT (https://chatgpt.com/, latest version available in July 2026; OpenAI, San Francisco, CA, USA), accessed in July 2026, on the basis of author-supplied layouts. No generative artificial intelligence tool was used to generate, analyse or interpret scientific content, to draft the text, or to select or summarise the cited literature. The authors reviewed and accept full responsibility for the content of the figures.

## 3. Curve Magnitude and Pain: Evidence for Dissociation

In adolescents, a major limitation of the available literature is that many AIS studies report the broader endpoint of “back pain” without anatomical localization.

A systematic review specifically addressing LBP [15] reported a prevalence of 34.7–42.0%, but identified only two eligible studies, both at high risk of bias, and concluded that the evidence was insufficient for a confident estimate. Moreover, a large 2024 AIS cohort [17] showed that pain, when present, was most frequently localized to the lumbar region, particularly among patients with lumbar curves, and more robust longitudinal data showed that in 428 untreated AIS patients, incident back pain rose from 14.7% at one year to 19.0% at three years, most of it mild, while newly symptomatic patients scored significantly worse on three patient-reported domains of the SRS-22r (function, self-image and mental health) reflecting the impact of pain on the patient’s own perceived capacity, body image and psychological state, rather than on any objectifiable finding on imaging [18]. With regard to imaging findings, curve magnitude alone does not account for pain burden, as reported in the literature. In 272 patients stratified into high-pain (*n* = 101, mean Numeric Rating Scale—NRS—5.3) and low-pain (*n* = 171, mean NRS 1.1) groups, multivariate logistic regression identified age (OR 1.07), school or work absenteeism (OR 3.87), Hospital Anxiety and Depression Scale (HADS) anxiety subscale (OR 1.18) and an indication for surgery (OR 2.87) as independent predictors, while curve magnitude did not enter the model [3]. In 1442 Scandinavian patients, 32% reported back pain on SRS-22r and none of 37 candidate pain-susceptibility variants was associated with it [19]. These findings argue against using deformity severity alone to explain the symptom burden.

Kinesiophobia is also associated with pain burden and functional impairment. In a cohort of 275 patients the TSK-11 (Tampa Scale of Kinesiophobia) showed acceptable internal consistency and no correlation with curve magnitude or Lenke pattern, indicating that movement-related fear is independent not only of curve severity but also of curve type (e.g., thoracic-only, thoracolumbar, or double curve), with significant but weaker correlations to pain and disability in adolescents than in young adults [20]; a subsequent controlled study of 128 participants confirmed correlation with pain intensity and absence of correlation with Cobb angle [21].

In ADS, this dissociation between imaging findings and the patient’s pain experience becomes even more pronounced. In a cohort of 100 adults with chronic LBP and degenerative lumbar scoliosis stratified by convexity, pain drawings showed no association between curve structure and either pain severity or laterality; pain was predominantly central and lumbar irrespective of coronal deformity, leading the authors to classify LBP in this population as non-specific [6]. In ASD, sagittal alignment appears more consistently associated with disability than coronal curve magnitude, and the SRS–Schwab classification was constructed and validated around these parameters [12,22]. Even after surgery, persistent pain is predicted by preoperative pain intensity and mental health rather than by radiographic correction [23].

## 4. Potential Pain Generators, Pain Modifiers, and Associated Conditions in Scoliosis-Related LBP

If curve magnitude alone does not explain pain, the diagnostic task becomes identifying the most clinically plausible mechanism or contributor. Table 1 presents a seven-component framework. Five components are candidate pain generators, each anatomically localizable and each potentially amenable to a supportive diagnostic step: (1) facet-mediated pain; (2) vertebrogenic or endplate-mediated pain; (3) foraminal or lateral recess neural compression; (4) sagittal malalignment with paraspinal mechanical overload; and (5) sacroiliac joint overload. The remaining two components are not pain generators in the same sense and are deliberately distinguished from them: (6) biopsychosocial pain modifiers, which have no imaging correlate but influence symptom burden, disability and treatment response; and (7) associated neural-axis abnormalities, which represent alternative or concurrent neurological conditions requiring a different diagnostic and therapeutic pathway rather than a target for pain-directed intervention. This seven-component structure is used consistently in the abstract, in the text, and in Table 1. Potential pain generators frequently coexist, and diagnostic confidence depends on concordance among symptoms, examination, imaging and, for selected interventions, diagnostic blocks. The framework is a synthesis assembled by the authors from the mechanistic, imaging and interventional literature reviewed here. It has not undergone consensus development, external review or prospective validation, and it should be read as a hypothesis-generating conceptual model rather than as a validated diagnostic instrument. Imaging can support a candidate generator by demonstrating an anatomically concordant phenotype, but it cannot establish causation by itself.

Among the structural candidate pain generators, facet-mediated pain may arise from asymmetric loading of the posterior elements, the distribution of which varies with curve morphology, vertebral rotation, and local biomechanics. Degenerative changes, synovitis, capsular distension and subchondral abnormalities may provide nociceptive input through the medial branches of the dorsal rami. MRI and CT can demonstrate facet joint degeneration, effusion, periarticular edema, subchondral change, joint space narrowing and osteophytes. However, these findings are common in degenerative spines and are not sufficiently specific to establish a facet-mediated origin of pain; imaging should therefore be used to identify an anatomically plausible target and to exclude competing causes rather than as a stand-alone diagnostic test [8].

When medial branch radiofrequency ablation is considered, clinical concordance and a positive diagnostic medial branch block remain necessary to support the diagnosis and treatment selection [8]. Vertebrogenic pain represents a second potential structural mechanism and is associated with vertebral endplate damage, inflammatory remodeling and sensitization of the basivertebral nerve. Modic type 1 and type 2 endplate changes on MRI constitute the imaging phenotype used in basivertebral nerve ablation trials and current patient-selection pathways, but their presence should not be equated with proof that the endplate is the dominant pain source in an individual patient. Conversely, isolated Modic type 3 change falls outside the established trial and label-selection phenotype for basivertebral nerve ablation [10].

Neural compression represents a further clinically relevant structural mechanism, particularly in ADS, where vertebral rotation, rotatory subluxation and lateral listhesis may contribute to asymmetric foraminal or lateral recess narrowing [25]. MRI is the principal technique for defining the level, severity and mechanism of neural compromise, but imaging abnormalities become clinically meaningful only when they correspond to the distribution of radicular symptoms and neurological findings. Conventional supine MRI may also underestimate dynamically accentuated foraminal narrowing by removing gravitational and postural loading, an issue that should be considered when symptoms and conventional imaging are discordant [27].

Mechanical overload may contribute to LBP through mechanisms that are not easily attributable to a single anatomical structure. Sagittal malalignment, particularly PI–LL mismatch, may require compensatory pelvic retroversion and sustained paraspinal muscle activation to maintain an upright posture, thereby contributing to muscular fatigue and myofascial pain. Measures of sagittal alignment have been associated with disability more consistently than coronal Cobb angle in adult spinal deformity [22]. Paraspinal muscle degeneration may further contribute to this mechanical phenotype: fatty infiltration and atrophy can be quantified on cross-sectional MRI and have been associated with symptom severity and surgical outcome, although these changes remain associative imaging biomarkers rather than specific indicators of pain origin [12]. Altered coronal and sagittal mechanics may also redistribute load across the pelvis and sacroiliac joints. Nevertheless, degenerative sacroiliac-joint abnormalities are frequent and nonspecific, and their presence on MRI or CT should not by itself be interpreted as evidence of a sacroiliac pain generator. In suspected mechanically mediated sacroiliac pain, imaging primarily serves to exclude inflammatory or alternative pathology, whereas attribution of symptoms relies on clinical provocative testing and, when an interventional procedure is contemplated, may require a concordant diagnostic sacroiliac-joint injection [9].

Not all clinically relevant contributors to pain in scoliosis should be conceptualized as structural pain generators. Occult neural-axis abnormalities, including syringomyelia, Chiari malformation and spinal cord tethering, may produce axial pain, radicular symptoms or myelopathic manifestations and should therefore be regarded as associated neurological conditions or alternative mechanisms rather than as pain generators equivalent to facet or endplate nociception [28,29]. Their identification has different diagnostic and therapeutic implications, prompting neurological or neurosurgical assessment and influencing MRI selection in adolescents with atypical or neurological features as well as preoperative neural-axis assessment [30]. Biopsychosocial factors may also modify symptom perception and disability independently of structural deformity. Among these factors, kinesiophobia has been directly investigated in scoliosis and has been associated with pain and functional impairment without a consistent relationship to Cobb angle or curve pattern [20,21]. These modifiers do not have a specific imaging correlate, but their recognition is important because they may influence symptom burden, treatment response and adherence to rehabilitation. Taken together, these observations support a multimodal interpretation of scoliosis-related LBP in which imaging identifies anatomically plausible structural, neural and mechanical contributors, while clinical assessment establishes concordance and recognizes non-structural factors capable of modifying the pain experience. Figure 1 represents a schematic overview of contributors to scoliosis-related LBP.

## 5. Imaging Strategy and Radiologist’s Role

In an LBP-focused approach, imaging has two complementary but distinct tasks: to characterize scoliosis and global alignment as part of the mechanical context, and to identify clinically plausible pain mechanisms or associated alternative conditions. Structural deformity and symptom origin should therefore not be considered interchangeable.

Standing full-spine radiography remains the reference examination for structural assessment. Baseline evaluation generally includes posteroanterior and lateral acquisitions, whereas subsequent radiographic follow-up may be tailored to the clinical question and the need for sagittal reassessment, with rotatory subluxation and lateral listhesis representing particularly relevant features in adult degenerative scoliosis [25]. Weight-bearing acquisition is important because spinal alignment may differ substantially between standing and supine positions, with supine imaging potentially underestimating components of the deformity [31]. Radiographic assessment should define curve magnitude and pattern together with global coronal and sagittal alignment. In adolescents, reports should include the Cobb angle with the end vertebrae specified, skeletal maturity, curve pattern, and coronal balance; in adults, assessment should additionally incorporate thoracic kyphosis, lumbar lordosis, pelvic incidence, pelvic tilt, sacral slope, sagittal vertical axis, PI–LL mismatch, and lateral listhesis [22].

MRI has a different role, and its indication should be driven by the clinical scenario. In adults with uncomplicated axial LBP, scoliosis alone is not an indication for routine MRI. Current appropriateness criteria support lumbar MRI principally after approximately six weeks of medical management and physical therapy with little or no improvement, or earlier when red flags are present, including cauda equina syndrome, suspected malignancy, fracture, infection, or a progressive or disabling neurological deficit [32]. When MRI is indicated in adults, a dedicated lumbar protocol should allow systematic assessment of the vertebral endplates, intervertebral discs, facet joints, central canal, lateral recesses, neural foramina, and paraspinal musculature. A practical protocol includes sagittal T1-weighted and fluid-sensitive T2-weighted imaging, preferably with fat suppression for evaluation of marrow and endplate abnormalities, together with axial T2-weighted acquisitions through the lumbar levels; additional axial T1-weighted or other targeted sequences may be incorporated according to the clinical question. This approach is consistent with the standardized acquisition and structured evaluation framework proposed by the BACPAC Spine Imaging Working Group for MRI phenotyping of LBP [33].

In adult scoliosis, sequence planning should account for vertebral rotation and asymmetric foraminal orientation. Axial images should therefore be prescribed according to the individual disc or endplate plane when necessary, with additional coronal or oblique acquisitions considered when standard planes do not adequately depict multilevel deformity or neural compromise. MRI should characterize Modic endplate changes, facet abnormalities, central and subarticular stenosis, foraminal compromise, nerve-root impingement, and paraspinal fatty infiltration or atrophy, while avoiding attribution of symptoms to these findings in isolation [9,10].

Intravenous gadolinium is not routinely required for degenerative scoliosis-related LBP and should be reserved for a specific clinical indication, such as suspected neoplastic, infectious, inflammatory or selected postoperative pathology. Standard MRI is acquired supine; when radicular or claudicant symptoms are substantially discordant with conventional imaging, dynamic effects should be considered, since axial loading has been shown to alter lumbar foraminal dimensions and may increase the apparent grade of foraminal compromise [27].

Whole-neuraxis MRI in adolescents serves a different purpose. In AIS with otherwise typical clinical and radiographic features, LBP alone does not mandate routine MRI; MRI becomes relevant when pain is associated with neurological findings or atypical clinical or radiographic features, and it is also commonly incorporated into the preoperative work-up for AIS correction as part of neurological risk assessment. A meta-analysis of 18 studies including 4746 patients reported a pooled prevalence of neural-axis abnormalities of 8% (95% CI, 6–12%), with approximately one third of affected patients with available management data undergoing neurosurgical treatment before scoliosis correction [26]. Subsequent surgical cohorts reported similar yields of 7.4% and 9.4% [28,29], whereas Johnson et al. found no neural-axis abnormalities among patients imaged solely for back pain with non-surgical curves [34]. Preoperative MRI and intraoperative neuromonitoring therefore provide complementary rather than interchangeable information [30]. These data support a low threshold for MRI in adolescents with atypical or neurological features and explain its use in many preoperative pathways, but they do not establish universal MRI as an investigation for uncomplicated scoliosis-related LBP.

In adolescents requiring MRI for scoliosis, the examination should instead be tailored to the detection of associated neural-axis and congenital abnormalities. Contextualized pediatric spine protocols include sagittal T1- and T2-weighted imaging of the relevant spinal axis, with targeted axial sequences for suspected cord, conus, or other focal abnormalities; Kadom et al. additionally recommend a coronal fat-suppressed T2-weighted sequence in scoliosis, to improve assessment of vertebral segmentation abnormalities and of the overall deformity [35].

When whole-neuraxis assessment is clinically indicated, coverage should include the craniocervical junction and entire spinal cord through the conus and lumbosacral region, allowing evaluation for Chiari malformation, syringomyelia, tethering, and other neural-axis abnormalities. Targeted axial T2-weighted imaging should be added across any suspected cord abnormality, and additional sequences should be tailored to the specific finding. Routine contrast administration is not required for uncomplicated scoliosis assessment and should be reserved for indications such as suspected tumour, infection, or inflammatory disease [35].

Structured reporting is the next essential component of the imaging pathway. In a series of 80 AIS radiographic reports, agreement between radiologists and spine surgeons was suboptimal, with an overall ICC of 0.65 and an ICC of 0.45 for community radiologists; Risser stage was omitted from 56 reports and absolute agreement on end-vertebra selection was achieved in only 23 cases [36]. Because differences of approximately 5° in Cobb measurement may influence assessment of progression and end-vertebra selection represents an important source of variability, reports should explicitly identify the end vertebrae rather than provide an isolated Cobb value. Automated Cobb-angle tools may improve reproducibility, but currently available systems remain predominantly retrospectively validated and should complement rather than replace expert assessment [37,38].

Radiation optimisation remains particularly important in adolescents undergoing serial monitoring. Breast tissue represents a major contributor to cumulative radiation risk in scoliosis imaging [39]. EOS biplanar slot-scanning radiography permits simultaneous weight-bearing frontal and lateral acquisition at substantially lower doses than conventional digital radiography; in a phantom study, microdose EOS yielded an effective dose of 2.6 ± 0.5 μSv compared with 67.5 ± 23.3 μSv for standard digital radiography [40]. Where EOS is unavailable, posteroanterior rather than anteroposterior acquisition provides a simple and substantial dose reduction, with reported breast-dose reductions of 76.7–89.1% in children and 80.4–91.1% in adults [41]. MRI-generated synthetic CT may provide radiation-free three-dimensional osseous information in selected pediatric applications, while photon-counting CT and deep-learning reconstruction represent additional developing dose-reduction strategies [42,43].

Finally, the radiologist’s role extends beyond acquisition and measurement. Imaging reports should distinguish deformity severity from the likely clinical relevance of individual abnormalities and avoid language implying that curve magnitude, Modic change, facet degeneration or stenosis necessarily explains the patient’s pain. Potential structural or neural contributors should instead be described anatomically and interpreted in the context of clinical concordance, leaving diagnostic blocks or other procedural tests to support, rather than establish, the symptomatic relevance of selected targets. This distinction is particularly important because anxiety, absenteeism and kinesiophobia have been associated with pain burden without a consistent relationship to curve magnitude [3,20]. Clear communication of structural findings, clinically plausible mechanisms and diagnostic uncertainty can therefore support multidisciplinary management without reinforcing an exclusively structural interpretation of scoliosis-related LBP. Table 2 summarises the imaging strategy according to clinical scenario.

## 6. From Imaging Phenotype to Low Back Pain Management

In adolescents with AIS and LBP, symptom management should be distinguished from treatment aimed at controlling deformity progression. Bracing is supported by randomized evidence for preventing curve progression in skeletally immature AIS, but the BrAIST trial was designed around progression to the surgical threshold rather than LBP relief: among 242 patients aged 10–15 years with Risser 0–2 and Cobb angles of 20–40°, treatment success was substantially higher with bracing than with observation, with a clear dose–response to wear time [44]. SOSORT recommendations and subsequent meta-analysis likewise support bracing primarily as a deformity-directed intervention [45,46]. Brace prescription should therefore be based on skeletal maturity and curve-progression risk, with any improvement in LBP regarded as a secondary clinical outcome.

Physiotherapeutic scoliosis-specific exercises (PSSE) are more directly relevant to symptom and functional management, although pain outcomes are frequently broader than anatomically defined LBP. The most comprehensive synthesis available, a Cochrane review of 13 randomised trials including 583 participants, judged the evidence on therapeutic exercise sparse and heterogeneous; back pain was only a minor outcome, and the review authors identified it among the endpoints that future trials should address [14]. A network meta-analysis of 17 RCTs including 857 patients reported improvements in Cobb angle, trunk rotation and quality of life compared with conventional rehabilitation [47], while a meta-analysis of nine RCTs including 571 patients found improvements in SRS-22 pain, function and self-image [48]. However, the certainty and durability of these effects remain limited: a Schroth-specific meta-analysis found no significant improvement in trunk rotation and a mean Cobb change below the conventional 5° threshold [49], while another systematic review identified no eligible medium-term study at low risk of bias and judged long-term certainty to be extremely low [50]. Adherence appears relevant, although available compliance data predominantly concern curve response rather than LBP-specific improvement [51]. PSSE may therefore be incorporated into conservative management of symptomatic adolescents without assuming that radiographic improvement represents improvement in LBP. The converse should also be stated. Although deformity-directed treatment in AIS is not an LBP-directed treatment, curve control is not irrelevant to long-term pain: the natural-history evidence that untreated late-onset idiopathic scoliosis is associated with more chronic back pain in the sixth decade than in matched controls [7] provides a coherent, if indirect, rationale for preventing progression to large residual curves in skeletally immature patients. What that evidence does not support is the use of bracing or exercise as a treatment for current pain, or the expectation that radiographic improvement will translate into symptomatic relief over the short term.

In adults, conservative care remains first-line, while interventional treatment should be reserved for clinically concordant and appropriately selected pain mechanisms. Structured multimodal physiotherapy has been associated with significant improvements in Visual Analog Scale (VAS) and Roland–Morris scores in adult degenerative scoliosis, sustained at three months [52]. Bracing may be considered in selected symptomatic adults, although evidence for pain relief remains limited and adherence may be affected by discomfort and aesthetic burden [53]. Predictors of achieving a minimal clinically important difference with non-operative treatment have included lateral flexion < 25°, trunk shift > 14 mm, pelvic incidence > 50° and baseline SRS-22r < 3.5, whereas coronal Cobb angle was not predictive [54]. Overall evidence remains weak: a systematic review found predominantly observational studies at very high risk of bias and no significant quality-of-life improvement at two years [55], while a three-arm RCT protocol involving 300 patients has been published [16]. Pharmacological treatment should therefore follow general guideline-based LBP principles and the dominant clinical pain phenotype rather than scoliosis-specific imaging findings [56].

Evidence for mechanism-directed interventions in patients with scoliosis is largely extrapolated from broader chronic LBP populations; therefore, scoliosis itself should not be considered an indication for these procedures, which require the same clinical, imaging, and diagnostic criteria applied in the general LBP population. For concordant radicular pain related to foraminal or lateral recess stenosis, epidural or selective nerve-root injection may provide temporary relief in selected patients, although benefit may decline over longer follow-up [57]. Suspected facet-mediated pain may be treated with medial branch radiofrequency ablation after a concordant diagnostic medial branch block [8]. For vertebrogenic pain, randomised evidence supports basivertebral nerve ablation in patients with chronic axial LBP and Modic type 1 or 2 changes at L3–S1, with a mean ODI difference of 20.3 points versus standard care in 140 patients [24], above the 12.8-point minimum clinically important difference [58].

These figures derive from non-scoliotic Modic-positive cohorts; the procedure is untested in scoliosis, and reported extension to osteoporosis, multilevel Modic change and adult spinal deformity lies outside the established evidence base [10].

Deformity correction becomes relevant when LBP and disability coexist with accepted deformity-related indications, including progression, major coronal or sagittal imbalance, neurological compromise, or persistent functional limitation despite appropriate conservative treatment.

The largest prospective comparative study in this setting is the Adult Symptomatic Lumbar Scoliosis (ASLS-1) study, conducted at nine North American centres in patients aged 40–80 years with a lumbar Cobb angle ≥ 30° and either ODI ≥ 20 or an SRS-22 subscore ≤ 4.0. It enrolled concurrent randomized (*n* = 63) and observational (*n* = 223) cohorts. In the randomized cohort, 64% of patients allocated to non-operative care crossed over to surgery, and the intention-to-treat comparison showed no difference between groups at two years; the reported treatment effects therefore derive from as-treated analyses, in which treatment reflected patient choice rather than random allocation. Revision surgery was required in 14% of operative patients within two years [59]. In the combined as-treated analysis at five years (179 operative and 107 non-operative patients; 74% follow-up), operative treatment was associated with greater improvement in ODI (mean difference −15.2 points, 95% CI −18.7 to −11.7) and in the SRS-22 subscore (+0.63, 95% CI 0.48–0.78), both exceeding the minimum detectable measurement difference, while treatment-related serious adverse events occurred at 22.38 per 100 person-years in the first two postoperative years and 8.17 per 100 person-years thereafter [60]. Because as-treated comparisons are susceptible to selection bias and residual confounding, and approximately one quarter of patients were not followed up at five years, these estimates indicate an association rather than a randomized treatment effect.

In 28 young adults aged 18–40 years with unoperated AdIS, posterior fusion reduced the maximum coronal curve from 43° to 17° and was accompanied by improvements in ODI (31 to 24), VAS back pain (6.0 to 4.0) and self-image, although the complication rate was 18% [61].

The 7-point ODI change falls below the 12.8-point minimum clinically important difference established for lumbar spine surgery [58], and the series was uncontrolled and reported no reoperation data; it therefore indicates a plausible direction of effect rather than established efficacy.

In adult spinal deformity, the extent of correction and the use of osteotomy are important determinants of perioperative risk [62], requiring potential pain and functional benefits to be weighed against surgical burden. Age-adjusted alignment targets have been proposed to reduce the risk of inappropriate over- or under-correction [63]; detailed deformity-correction strategies, however, remain beyond the scope of this LBP-focused review. Figure 2 presents an author-proposed conceptual pathway for scoliosis-related LBP in adolescents and adults.

Table 3 sets out the principal conclusions of this review against the evidence supporting each. For every conclusion it records the supporting studies and their design, the outcome actually measured, and whether the conclusion rests on scoliosis-specific data or is extrapolated; it describes the evidence rather than grading its certainty, as no formal certainty assessment was performed. It is intended to make explicit where the argument developed here is empirically grounded and where it remains inferential.

## 7. Conclusions

Pain in scoliosis is not explained by curve magnitude alone. Imaging can identify clinically plausible structural, neural, and mechanical contributors, but these findings do not establish symptom causation; attribution of a pain source therefore requires clinical and radiological concordance and, for selected interventions, supportive diagnostic procedures. Biopsychosocial factors may further modify symptom burden, while occult neural-axis abnormalities should be considered associated or alternative neurological conditions rather than equivalent structural pain generators.

The practical implications differ between adolescents and adults. In adolescents with AIS and LBP, structural deformity assessment should be distinguished from the search for associated neural-axis pathology, and deformity-directed treatment should not be assumed to provide LBP-specific benefit. Whole-neuraxis MRI is particularly relevant in atypical, neurological, and preoperative settings, but available evidence does not support its routine use for uncomplicated scoliosis-related LBP. In adults, the curve remains potentially modifiable when deformity correction is otherwise indicated, but pain management should primarily target clinically concordant and treatable mechanisms rather than deformity magnitude alone.

These conclusions should be interpreted in light of the narrative design of this review, including single-reviewer study selection and the absence of formal risk-of-bias or certainty-of-evidence assessment (Section 2). The proposed seven-component framework and the management pathway of Figure 2 are author-derived conceptual models that have not undergone consensus development or prospective validation. Much of the AIS literature reports the broader endpoint of “back pain” without anatomical localization, and several conclusions rest on disability, quality-of-life or radiographic outcomes rather than on anatomically defined LBP; Table 3 identifies which conclusions are supported by LBP-specific data and which are extrapolated from non-scoliotic chronic LBP cohorts. The adult non-operative literature remains of limited quality, and cumulative radiation exposure during serial monitoring in adolescents, although addressed here, has not been evaluated against pain-related outcomes. Future research should prioritize prospective validation of mechanism-directed treatment pathways, randomized studies of conservative management in adults with scoliosis-related LBP, adolescent studies using anatomically defined LBP outcomes, and systematic integration of biopsychosocial phenotyping with structural imaging assessment.

## Figures and Tables

**Table 1:** Author-proposed seven-component framework of candidate pain generators, pain modifiers and associated conditions in scoliosis-related low back pain. The framework is author-derived and has not been prospectively validated. Imaging findings identify anatomically plausible contributors and do not establish pain causation; interpretation requires clinical concordance and, for selected interventions, supportive diagnostic procedures. The “Evidence base” column indicates whether the supporting evidence is scoliosis-specific or extrapolated from non-scoliotic chronic LBP cohorts. Abbreviations: BVNA, basivertebral nerve ablation; CBT, cognitive behavioural therapy; HADS, Hospital Anxiety and Depression Scale; PCS, Pain Catastrophising Scale; PI–LL, pelvic incidence minus lumbar lordosis; PT, pelvic tilt; RFA, radiofrequency ablation; SI, sacroiliac; SRS-22r, refined Scoliosis Research Society 22-item questionnaire; SVA, sagittal vertical axis; TSK-11, 11-item Tampa Scale of Kinesiophobia.

	Component	Key Imaging Findings	Concordance/Supportive Diagnostic Step	Management Implication	Evidence Base
1	Facet-mediated pain	Facet degeneration, effusion, subchondral oedema, joint narrowing/osteophytes	Concordant axial pain distribution + positive diagnostic medial branch block	RFA after positive block	Extrapolated from non-scoliotic chronic LBP; guideline-level [8]
2	Vertebrogenic/endplate pain	Modic type 1 or 2 change, L3–S1	Chronic axial LBP phenotype + exclusion of alternative sources; imaging alone not diagnostic	Conservative care; BVNA in eligible refractory patients	Extrapolated; RCT-level in non-scoliotic Modic-positive cohorts [24]
3	Foraminal/lateral recess compression	Foraminal narrowing, root impingement, lateral listhesis, rotatory subluxation	Concordant radicular distribution + neurological findings; selective root block where equivocal	Root block/epidural injection; decompression when indicated	Partly scoliosis-specific (ADS anatomy) [25]; therapeutic evidence extrapolated
4	Sagittal malalignment/paraspinal overload	SVA, PI–LL mismatch, PT; paraspinal fatty infiltration and atrophy	Mechanical/postural symptom pattern assessed in global sagittal context	Rehabilitation; deformity correction in selected ASD	Scoliosis-specific, observational; ASD-derived [22]
5	SI-joint overload	Degenerative SI change; principal role is exclusion of alternative pathology	Provocative testing ± concordant diagnostic SI injection	Targeted injection/RFA in selected cases	Extrapolated; no scoliosis-specific data [9]
6	Biopsychosocial pain modifiers	No imaging correlate	TSK-11, PCS, HADS; clinical assessment	Education, graded rehabilitation, CBT-based approaches	Scoliosis-specific, observational [20,21]
7	Associated neural-axis abnormalities	Syrinx, Chiari malformation, tethering, cord abnormalities	Neurological or atypical clinical context; whole-neuraxis MRI	Neurological/neurosurgical assessment—not a pain-directed target	Scoliosis-specific, meta-analytic prevalence [26]

**Table 2:** Imaging strategy for scoliosis-related low back pain in adolescents and adults according to clinical scenario, including first-line imaging, MRI indications, and key reporting parameters.

Clinical Scenario	First-Line Imaging	MRI Indication	Key Reporting Parameters
AIS with LBP, no atypical features	PA standing full-spine radiograph; low-dose biplanar EOS when available	Not routinely indicated for LBP alone	Cobb angle and end vertebrae; skeletal maturity; curve pattern; coronal balance
AIS with LBP, surgical planning	PA and lateral standing full-spine radiographs; low-dose biplanar EOS when available; bending views as required	Whole-neuraxis MRI commonly included in preoperative assessment	Structural and sagittal parameters; neural-axis abnormalities
AIS with LBP plus atypical or neurological features	PA and lateral standing full-spine radiographs; low-dose biplanar EOS when available	Whole-neuraxis MRI	Structural parameters; Chiari malformation; syringomyelia; tethered/split cord; other neural-axis abnormalities
Adult scoliosis with axial LBP	PA and lateral standing full-spine radiographs	Persistent/progressive symptoms despite conservative care; red flags; neurological features; intervention/surgical planning	If MRI obtained: Modic phenotype; facets; central/lateral recess/foraminal stenosis; paraspinal muscles; alternative pathology
Adult scoliosis with radiculopathy or claudication	Standing full-spine radiographs + targeted lumbar assessment	Persistent/progressive symptoms when intervention/surgery is contemplated; earlier for red flags or progressive neurological deficit	Rotatory subluxation/lateral listhesis; level-specific foraminal or lateral recess stenosis; neural compression

**Table 3:** Principal conclusions of this review, with supporting evidence, outcome measured, and evidentiary basis. The final column describes the design and applicability of the supporting evidence; no formal certainty-of-evidence grading was performed. “Scoliosis-specific” indicates that the supporting study enrolled patients with scoliosis; “extrapolated” indicates that the evidence derives from chronic LBP cohorts in which scoliosis was not an inclusion criterion. AdIS, adult idiopathic scoliosis; ADS, adult degenerative scoliosis; AIS, adolescent idiopathic scoliosis; ASD, adult spinal deformity; ATR, angle of trunk rotation; MCID, minimum clinically important difference; MDMD, minimum detectable measurement difference. HRQoL, health-related quality of life; ODI, Oswestry Disability Index; RCT, randomized controlled trial; SRS-22, Scoliosis Research Society 22-item questionnaire; TSK-11, 11-item Tampa Scale of Kinesiophobia.

Conclusion	Evidence	Outcome Measured	Basis and Applicability
Curve magnitude is not an independent predictor of pain burden in AIS	Cross-sectional cohorts, *n* = 272 and *n* = 1442 [3,19]	Pain intensity; SRS-22r back pain	Scoliosis-specific; two observational cohorts
Pain distribution does not follow curve laterality in ADS	Cross-sectional, *n* = 100, pain drawings [6]	Pain location and severity	Scoliosis-specific; single cross-sectional study
LBP prevalence in AIS cannot be confidently estimated	Systematic review, 2 eligible studies, both high risk of bias [15]	Anatomically defined LBP	Scoliosis-specific; two eligible studies, both at high risk of bias
Biopsychosocial factors relate to pain burden independently of curve magnitude	Cross-sectional, *n* = 275 and *n* = 128 [20,21]	TSK-11; pain; disability	Scoliosis-specific; two cross-sectional studies
Sagittal alignment relates to disability more consistently than coronal Cobb angle	SRS–Schwab development and validation [22]	ODI; SRS-22; HRQoL	Scoliosis-specific (ASD); classification development and validation
Neural-axis abnormalities occur in ~8% of presumed AIS	Meta-analysis, 18 studies, *n* = 4746 [26]	Prevalence of MRI abnormality	Scoliosis-specific; pooled prevalence from observational studies
MRI is not indicated for uncomplicated axial LBP before ~6 weeks, absent red flags	Appropriateness criteria [32]	Imaging appropriateness	Extrapolated; guideline recommendation for general LBP
Deformity-directed treatment in AIS is not LBP-directed treatment	RCT, *n* = 242 BrAIST [44]; Cochrane review, 13 RCTs, *n* = 583 [14]	Curve progression; Cobb, ATR, SRS-22—back pain only a minor outcome	Scoliosis-specific; randomized evidence for curve progression only, no LBP-specific outcome
Mechanism-directed interventions rest on evidence from non-scoliotic populations	Guideline [8]; RCT, *n* = 140, Modic 1/2 [24]; ODI MCID 12.8 [58]	Pain; ODI (−20.3, above MCID)	Extrapolated; randomized and guideline evidence from non-scoliotic cohorts, untested in scoliosis
Deformity correction is associated with greater improvement where disability and malalignment coexist, but not demonstrably in young adults	Prospective multicentre, *n* = 286, 5 years [59,60]; uncontrolled series, *n* = 28 [61]	ODI −15.2 and SRS-22 +0.63, both > MDMD; vs. ODI 31→24, below MCID, 18% complications	Scoliosis-specific; ASLS: as-treated comparison of combined randomized and observational cohorts, susceptible to selection bias; AdIS: uncontrolled series

## Data Availability

No new data were created or analyzed in this study. Data sharing is not applicable to this article.

## Abbreviations

The following abbreviations are used in this manuscript:

Abbreviation	Full term
AIS	Adolescent Idiopathic Scoliosis
BACPAC	Back Pain Consortium Research Program
BrAIST	Bracing in Adolescent Idiopathic Scoliosis Trial
BVNA	Basivertebral Nerve Ablation
CBT	Cognitive Behavioural Therapy
CI	Confidence Interval
CT	Computed Tomography
EOS	Biplanar slot-scanning radiography system
AdIS	Adult Idiopathic Scoliosis
ADS	Adult Degenerative Scoliosis
ASD	Adult Spinal Deformity
ASLS	Adult Symptomatic Lumbar Scoliosis
ATR	Angle of Trunk Rotation
CENTRAL	Cochrane Central Register of Controlled Trials
HRQoL	Health-Related Quality of Life
MDMD	Minimum Detectable Measurement Difference
SRS	Scoliosis Research Society
HADS	Hospital Anxiety and Depression Scale
ICC	Intraclass Correlation Coefficient
LBP	Low Back Pain
LL	Lumbar Lordosis
MCID	Minimal Clinically Important Difference
MRI	Magnetic Resonance Imaging
NRS	Numeric Rating Scale
ODI	Oswestry Disability Index
OR	Odds Ratio
PA	Posteroanterior
PCS	Pain Catastrophising Scale
PI	Pelvic Incidence
PI-LL	Pelvic Incidence minus Lumbar Lordosis
PSSE	Physiotherapeutic Scoliosis-Specific Exercises
PT	Pelvic Tilt
RCT	Randomised Controlled Trial
RFA	Radiofrequency Ablation
SANRA	Scale for the Assessment of Narrative Review Articles
SI	Sacroiliac
SOSORT	Society on Scoliosis Orthopaedic and Rehabilitation Treatment
SRS-22r	Refined Scoliosis Research Society 22-item questionnaire
SS	Sacral Slope
SVA	Sagittal Vertical Axis
T1	T1-weighted (imaging sequence)
T2	T2-weighted (imaging sequence)
TSK-11	11-item Tampa Scale of Kinesiophobia
VAS	Visual Analogue Scale
